# Prevalence of iodine deficiency among Moroccan women of reproductive age

**DOI:** 10.1186/s13690-022-00901-7

**Published:** 2022-05-27

**Authors:** Anass Rami, Naima Saeid, Mohammed El Mzibri, Khalid El Kari, Mohamed Idrissi, Houria Lahmam, Fatima Zahra Mouzouni, Samir Mounach, Laila El Ammari, Hasnae Benkirane, Ayoub Al Jawaldeh, Micheal Bruce Zimmermann, Hassan Aguenaou

**Affiliations:** 1 Laboratory of Biology, Health and Environment, Ibn Tofail University, Kenitra, Morocco; 2 Nutrition and Alimentation Unit, CNESTEN, Rabat, Morocco; 3 Ministry of Health, Rabat, Morocco; 4 World Health Organization – Regional Office for Eastern Mediterranean, P.O. Box 7608, Nasr City, Cairo Egypt; 5 ETH Zürich, Laboratory of Human Nutrition, Institute of Food Nutrition and Health, Department of Health Science and Technology, ETH Zürich, Zürich, Switzerland

**Keywords:** Urinary Iοdine, Mοrοccο, Iodine-rich food, Wοmen Reprοductive age

## Abstract

**Background:**

Iodine deficiency disorders (IDD) affects nearly 1.9 million people worldwide. Iodine deficiency (ID) remains a public health concern not only for pregnant women, but for women of reproductive age (WRA) as well. This study was planned to evaluate the iodine status and the prevalence of iodine deficiency in a nationally representative sample of Moroccan WRA according to their socio-economic data and living areas.

**Methods:**

This study is a cross-sectional national survey conducted on 1652 WRA aged between 18 and 49 years. Iodine status was assessed by the evaluation of the urinary iodine concentration (UIC) on spot urinary samples, using the Sandell-Kolthoff reaction, and by the estimation of iodine-rich food consumption, using a food frequency questionnaire. The World Health Organization cutoff of a median UIC of < 100 μg/l was used to define ID in the population.

**Results:**

The median UIC [20th- 80th] was 71.3 μg/l [37.5–123.1] and 71% of participants had UIC < 100 μg/L, indicating insufficient iodine status and mild iodine deficiency. WRA from urban and rural areas showed an UIC median of 75.94 μg/l [41.16–129.97] and 63.40 μg/l [33.81–111.68], respectively. Furthermore, ID prevalence was significantly higher in rural areas (75.6%) as compared to urban areas (67.9%) (*p* < 0.05). Food frequency questionnaires analyses highlighted that dairy products are the most commonly consumed iodine-rich food, reported to be consumed daily by 43.1% of WRA. Of particular interest, 83.5% of WRA reported a weekly consumption of fish.

**Conclusion:**

ID is still a public health problem in Morocco highlighting the necessity to implement effective national program, including efficient salt iodization, effective nutritional education and awareness, to control iodine deficiency and prevent IDD development.

## Background

Iodine is an essential trace element indispensable for thyroid hormone production, including thyroxine (T4) and triiodothyronine (T3), required for normal growth and involved in various pathways of metabolism [1]. Thyroid-associated diseases are among the most common endocrine disorders, including too much (hyperthyroidism) or too little (hypothyroidism) thyroid hormones. Thyroid hormones are of fundamental importance for normal brain development [2], and accordingly, human fetus, newborns, and young children are particularly vulnerable to iodine deficiency [3].

Worldwide, iodine deficiency affects more women than men and is more pronounced in women of reproductive age (WRA), affecting drastically the growth and health of newborns.

Indeed, during pregnancy, thyroid failure exerts destructive impacts on maternal metabolism and embryonic growth, leading to less weight at birth and congenital hypothyroidism [4, 5].

In humans, iodine is present at very low quantities (10–15 mg) that are closely affected by daily iodine intake. Accordingly, Iodine deficiency occurs when iodine intake falls below recommended levels (150 μg/ day) [6], leading to low thyroid hormones production, widely reported to be the major cause of a spectrum of diseases and a variety of health problems and abnormalities, affecting all population categories, collectively classified as Iodine Deficiency Disorders (IDDs) [7, 8].

As a result, the WHO recommendation emphasizes the importance of consuming iodine-rich food, particularly during the prenatal period and the first trimester of pregnancy, for WRA and those considering pregnancy. Hence, to support all physiological and cellular needs of WRA, daily iodine intake must exceed 100 μg/day [9].

Iodine is naturally supplied through animals, plants grown on iodine-rich soils and seafood, including fish, mollusks and algae [10, 11]. The contribution of these iodine-rich dietary sources to the total iodine intake may be slightly overestimated due to an underestimation of the total iodine intake that include iodine from other resources like supplements, water or iodized salt, accounting for 10–20% of total iodine intake [12].

Iodine status can be estimated directly by the measurement of urinary excretion or indirectly using the 24 h dietary recall or any other recall which can quantify food intake [13]. Currently the urinary iodine (UI) is identified as potentially the most useful indicator of recent iodine intake [14]. Indeed, the 24-hour urinary iodine excretion (UIE) is considered as the gold standard method for iodine intake assessment [15]. However, the main limitation of this approach is the difficulty to collect the 24-hour urine sample and its unwieldiness for large-scale epidemiological studies [16, 17]. Thence, to overcome this limitation, the median urinary iodine concentration (UIC) measured on spot urine samples is the most commonly used biomarker to assess iodine intake in a population group and epidemiological studies [18–20].

Moreover, the iodine-creatinine ratio (I/Cr ratio) has been used apart from UIC (μg/l) to assess iodine status [21] and is considered an accurate approach to estimate the 24-hour UIE [22, 23]. Alternatively, dietary assessment methods can be used to assess the usual iodine intake, to identify major sources of dietary iodine and to assess iodine status through dietary recommendations [24]. Food Frequency Questionnaires (FFQ) are one of the most common dietary methods used to measure dietary intake in nutrition research to assess the habitual iodine consumption of iodine-rich food groups e.g. (milk and dairy products, fish, eggs) [25].

In Morocco, the diet is basically Mediterranean and is based on a large consumption of cereals, fruits, and vegetables [26]. Globally, Morocco is classified as having a moderate iodine deficiency [27], and according to the Iodine Global Network (IGN), WHO and UNICEF recommendations, Moroccan government implemented the salt iodization strategy in 1995 to control iodine deficiency and prevent IDDs. Unfortunately, results of performed studies and carried surveys converge towards a low iodine status among the Moroccan population which remains more pronounced in some regions of the country [28].

The present study was planned to evaluate the iodine status of a nationally representative sample of Moroccan WRA by Urinary Iodine Concentration (UIC) and Food Frequency Questionnaire (FFQ) as well as to assess the association between iodine deficiency and socio-economic data and living areas of WRA.

## Methods

### Study design

The present study is a part of the national survey on Non-Communicable Diseases (NCDs) risk factors, the STEPwise Survey-Morocco, conducted by the Moroccan Ministry of Health between 2017 and 2018. Very briefly, the survey used the WHO STEPwise guidelines [29] to collect nationally representative data from Moroccan adult aged (18 years and over) in all the 12 regions of the country, covering four geographical zones: (1) Coastal and mountainous zone regrouping the North and the east regions; (2) Coastal and non-mountainous zone regrouping areas limited to the West region; (3) Non-coastal with high altitude characterizing the Centre of the country; and (4) Coastal, mountainous and desert zone regrouping all the southern regions. The field work was conducted between March and June 2017.

### Population

Participants in the STEPwise Survey-Morocco-2017-2018 were screened using the WHO STEPs sampling design [29]. Data from the 2014 National Census, the most recent one in Morocco, was used as sampling frame for the survey. First, primary units (units of more than 50 households) from urban and rural areas were randomly selected using a geographical stratification and each area has a specific stratification:

- For urban areas, the criteria used were the administrative division of each region, provinces/prefectures and the dominant type of habitat.
- For rural areas, the primary units were stratified according to the geographical criterion, and the type of relief.

Next, a stratified three-stage cluster sampling procedure (cluster, household, and individual) was used. Clusters (a unit of 25 households) were randomly selected from each primary unit using Kish method, and households were then randomly selected from each cluster. Using a computer program, one eligible WRA was randomly selected from each household.

Women that were: (i) not residents at the screened household for more than 06 months; (ii) with family or personal history of thyroid disease; (iii) under thyroid medications or with thyroid disease history, (iiii) in menstruating period during the collection of urine and breastfeeding woman on the day of collection, were excluded from the study.

### Anthropometry measurements

Weight and height measurements were performed and recorded by trained health professionals according to the WHO standard protocol and using calibrated instruments [30]. Measurements were conducted with minimal clothing and without shoes. Body weight was measured to the nearest 0.1 kg using an electronic scale (Seca GmbH and Co. KG). Height was measured to the nearest at 0.1 cm using a stadiometer (seca GmbH and Co. KG). Body Mass Index (BMI) was calculated as a ratio of weight (kg) to the squared height (m^2^) (kg/m^2^).

### Food frequency questionnaire (FFQ)

In this study, an adapted FFQ was used to record all iodine-rich food consumed by the participants and the frequency of their consumption during the month before the survey.

Special mention has been given to the frequency of daily, weekly and monthly consumption of these iodine-rich foods. Thus, face-to-face interviews were conducted with all WRA to complete the FFQs with respect to the Stepwise surveys standard protocols [31].

### Spot urine collection

Each eligible WRA has received detailed oral and written instructions, to ensure the best conditions for urine collection and storage, and all material needed for the collection of spot urine. According to the STEPwise-Morocco-2017-2018 survey design, participants were invited to collect the evening spot urine (between 8 p.m. and 10 p.m.) and to store collected urine samples in a cool and dark place (in the freezer at least − 4 °C) [31]. Urine samples were then collected from households the next morning at 8:00 am by trained laboratory technicians. Once in the laboratory, and after complete thawing, the urine volume was registered, and after rigorous shaking, two 5 ml aliquots were prepared from each urinary spot sample and stored at − 80 °C until analysis.

### Urinary analysis

The urinary iodine concentrations of spot urine samples were determined using the ammonium persulfate digestion method based on the Sandell-Kolthoff reaction [32].

Urinary iodine determination is carried out two steps, i.e. urine digestion at high temperature and iodine measurement in Sandell-Kolthoff reaction:

1 $${As}^{3+}+{I}^2\to {As}^{5+}+2{I}^{-}.$$

2 $$2{Ce}^{4+}+2{I}^{-}\to 2{Ce}^{3+}+{I}^2.$$

Arsenite in the presence of iodine reduces yellow-colored cericions to colorless cerous ions. Thus, the absorbance, measured by spectrophotometry at 430 nm, is inversely correlated with the concentration of urinary iodine.

The urinary excretion of creatinine was analyzed by the kinetic approach according to the Jaffé method using the Cobas C311 (Roche Diagnostic, Meylan - France) [33].

Urinary iodine analysis was performed in The Human Nutrition, ETH - Zürich Swiss Federal Institute of Technology, Zürich, Switzerland, that has successfully participated in the Program of Ensuring the Quality of Urinary Iodine Procedures (EQUIP), a free global service provided by the US Centers for Disease Control and Prevention and currently used to assist more than 126 iodine laboratories in more than 60 countries [34].

Quality control: Four external control samples, ranging between 10 and 400 μg/l, were measured quarterly and results were compared to ICP- MS analysis performed US CDC (R^2^ = 0.99: data available on request). External quality control was ensured by measuring two control urine samples that were added to each plate analyzed.

For creatinine assessment, the urinary measurements were duplicated for 15% of randomly selected sample, and results showed a low analytical imprecision with a coefficient of variation of 1.4%.

Criteria of UIC and iodine-creatinine ratio concentrations established by the Iodine Global Network (IGN) group were used. Iodine statutes were defined as follow: excessive ≥300 μg/l, above adequate 299–200 μg/l, adequate 199–100 μg/l, mild 99–50 μg/l, moderate 49–20 μg/l and severe deficiency < 20 μg/l.

All samples were analyzed in duplicate, and the limit of detection was 10^− 2^ μg/l.

### Statistical analysis

Urinary iodine concentration was assessed for all samples, adjusted by age, education level, geographical origin, BMI and monthly household income categories. Data were examined for normality using Kolmogorov-Smirnov test. Normally distributed data were presented as means ± standard deviations (SD) for continuous variables and percentages ([95% confidence interval (95% CI]) for categorical variables, and differences were calculated using parametric tests. Data that showed deviation from normality were presented as median [20th percentile, 80th percentile], and differences were calculated using non-parametric tests. Differences were considered significant when *p* < 0.05. All statistical analyses were performed using IBM SPSS (version 21).

### Ethical approval

The study protocol was approved by the Ethics Committee for Biomedical Research, Faculty of Medicine and Pharmacy of Rabat – Morocco (Ethical Approval number 248/2016), and informed consents were obtained from all participants. Moreover, the project was validated by the scientific, technical and advisory committee on nutrition (institutionalized by a decision of the Ministry of Health in 2017) and conducted with respect to the Declaration of Helsinki.

## Results

Overall, 1854 WRA were recruited from the 12 regions of Morocco. Among them, 202 were excluded (155 women did not provide their urine and 47 samples had invalid iodine measurements (UIC < 3 μg/l or UIC ≥1000 μg/l). The remaining 1652 WRA constituted the final population of the study. The mean age of recruited WRA was 34.8 ± 8.5, overall, (35.1 ± 8.5 in women from urban areas and 34.2 ± 8.5 in rural areas (*p* = 0.999)).

Socio-demographic characteristics of the studied population are reported in Table 1. Globally, 60% of WRA were from urban areas and 40% from rural areas. Overall, most of recruited WRA were married (78.5%) and illiterates (44.8%), and the majority have reported a household income less than 338 $. WRA were mostly recruited from zones 1 (33.8%) and 3 (30.6%). Women from zone 2 and 4 represent 22.9 and 12.7% of total WRA recruited, respectively.

**Table 1:** Socio-demographic characteristics of the studied population

	% [95%CI]	% [95%CI]	% [95%CI]	***p*** Value
	Overall (***N*** = 1652)	Urban (***N*** = 992)	Rural (***N*** = 660)	
**Age**				
18–29	29.2 [27.0–31.4]	27.7 [24.9–30.5]	31.4 [27.8–34.9]	
30–39	36.2 [33.9–38.5]	35.4 [32.5–38.5]	37.5 [33.8–41.1]	0.044
40–49	34.6 **[**32.3–36.9**]**	36.9 [33.9–39.9]	31.1 [27.5–34.6]	
**Matrimonial status**				
Single	15.9 **[**14.1–17.7**]**	16.9 [14.5–19.2]	14.2 [11.5–16.8]	
Married	78.5 [76.5–80.5]	77.2 [74.6–79.8]	80.5 [77.4–83.5]	0.404
Divorced	2.9 [2.1–3.7]	3.3 [2.1–4.4]	2.6 [1.3–3.8]	
Widow	2.7 **[**1.9–3.5**]**	2.6 [1.6–3.5]	2.7 [1.4–3.9]	
**Education level**				
No formal education	44.8 **[**42.4–47.2**]**	33.5 [30.5–36.4]	62.0 [58.3–65.7]	
Primary	24.8 **[**22.7–26.9**]**	23.6 [20.9–26.2]	26.5 [23.1–29.8]	0.001
Secondary	23.7 **[**21.6–25.7**]**	32.0 [29.1–34.9]	11.0 [8.6–13.4]	
Superior	6.7 **[**5.4–7.9**]**	10.9 [8.9–12.8]	0.50 [0.1–1.1]	
Household incomes*				
⩽338	79.6 **[**77.2–81.9**]**	73.5 [70.7–76.2]	91.0 [88.2–93.1]	
338–676	11.7 **[**9.8–13.6**]**	14.4 [12.2–16.5]	6.7 [4.8–8.6]	0.001
677–1128	3.2 **[**2.2–4.2**]**	4.1 [2.8–5.3]	1.5 [0.5–2.4]	
⩾ 1129	5.5 **[**1.6–3.5**]**	8.0 [6.3–9.7]	0.8 [0.1–1.4]	
**Geographical origin**				
Zone 1	33.8 **[**31.5–36.0**]**	37.8 [34.8–40.8]	27.9 [24.4–31.3]	
Zone 2	22.9 **[**20.8–24.9**]**	26.2 [23.4–28.9]	17.9 [15.0–20.8]	0.01
Zone 3	30.6 [28.3–32.8]	22.4 [19.8–24.9]	43.0 [39.2–46.8]	
Zone 4	12.7 [11.1–14.3]	13.6 [11.4–15.7]	11.2 [8.8–13.6]	

Values are percentage (95% confidence interval; 95%CI).

The chi-square test was used to compare percentages [95% CI] for different Socio-demographic categories between urban and rural areas.

* P value was calculated based on available data (*n* = 1118)

Comparison between socio-economic characteristics of WRA from urban and rural areas showed significant difference according to age, educational level, household income and geographical origin (*p* < 0.05).

Anthropometric characteristics of recruited WRA are reported in Table 2 and results clearly showed that most women were overweighed. Indeed, 38.7% of WRA have an overweight (639/1652) and 24.2% were obese (399/1652). Comparison of WRA anthropometric characteristics between rural and urban areas showed that obesity is significantly more prevalent in urban areas (*p* = 0.001) and normal weight is more pronounced in rural areas (*p* = 0.001).

**Table 2:** Anthropometric characteristics of the WRA

		Overall (***N*** = 1652)	Urban(***N*** = 992)	Rural (***N*** = 660)	***p***-Value
		Mean ± SD	Mean ± SD	Mean ± SD	
Age (years)		34.8 ± 8.5	35.1 ± 8.5	34.2 ± 8.5	0.999 ^a^
Height (cm)		160.2 ± 6.1	160.4 ± 6.0	159.3 ± 5.9	0.640 ^a^
Weight (kg)		68.6 ± 13.7	70.3 ± 13.9	66.2 ± 13.0	0.061 ^a^
BMI (Kg/m^2^)		26.7 ± 5.1	27.1 ± 5.2	26.1 ± 4.9	0.097 ^a^
Nutritional status°		N (%)	N (%)	N (%)	
BMI (Kg/m^2^)	Thinness	70 (4.2%)	41 (4.1%)	29 (4.4%)	0.689 ^b^
	Normal weight	544 (32.9%)	292 (29.5%)	252 (38.2%)	0.001 ^b^
	Overweight	639 (38.7%)	389 (39.2%)	250 (37.9%)	0.386 ^b^
	Obese	399 (24.2%)	270 (27.2%)	129 (19.5%)	0.001 ^b^

Values are mean (SD) and percentage [95% interval confidence (95%CI)]/ percentage.

Differences were calculated using interdependent t- test ^a^ and Pearson Chi-square test ^b^.

° Body Mass Index calculated as weight in kg divided by height in meter square (BMI; kg/m^2^) was used to define Nutritional status as follow: Thinness (< 18.5 kg/m^2^), normal weight (18.5─24.9 kg/m^2^), overweight (25.0─29.9 kg/m^2^) and obese (≥ 30.0 kg/m^2^).

Results of the urinary iodine assessment are summarized in Table 3. Overall, UIC [20th–80th] and iodine-creatinine ratio (I/CR ratio) medians were 71.3 μg/l [37.5–123.1] and 76.6 μg/g [43.9–146.1], respectively. Distribution of iodine status according to the WRA living area showed that women from urban areas had higher iodine status than their counterparts from rural areas and significant differences were observed for both UIC and I/CR ratio (*p* < 0.05).

**Table 3:** Median of UIC and iodine-to-creatinine ratio level of WRA according to living area

	Overall	Urban	Rural	p-Value^**a**^
	Median [20th–80th]	Median [20th–80th]	Median [20th–80th]	
UIC (μg/l)	71.3 [37.5–123.1]	75.9 [41.1–129.9]	63.4 [33.8–111.6]	0.0001
Creatinine Concentration (g/l)	0.93 [0.89–1.01]	0.97 [0.91–1.09]	0.86 [0.84–1.05]	0.044
I/Cr (μg/g)	76.6 [43.9–146.1]	78.1 [46.5–155.1]	73.6 [40.2–140.8]	0.01

^a^ calculated using Mann Whitney u test

*UIC* urinary iodine concentration, *I/Cr ratio* iodine/creatinine ratio

**Fig. 1:**
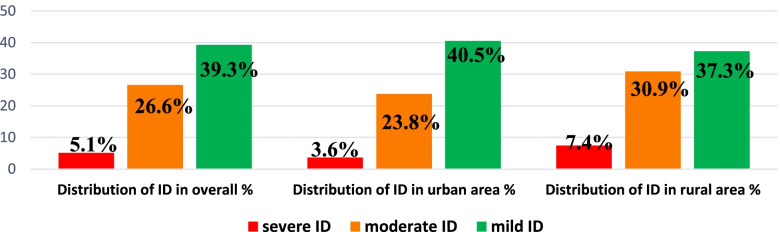
Distribution of percentages of women with mild, moderate and severe iodine deficiency (ID) status overall and by area of residence

In the present study, 71% of recruited WRA showed iodine deficiency (1173/1652). Among them, 5.1% showed severe iodine deficiency (84/1652), moderate and mild iodine deficiencies were reported in 26.6% (440/1652) and 39.3% (649/1652), respectively (Fig. 1). Distribution of iodine deficiencies according to living area showed a significant difference between rural and urban areas (*p* < 0.05). In Urban area, 67.9% of WRA had iodine deficiency, mainly a mild and moderate deficiencies, reported in 40.5 and 23.8%, respectively. In contrast, rural area registered 75.6% of deficient WRA and the prevalence of mild and moderate iodine deficiencies were 37.3 and 30.9%, respectively. Of particular interest, severe iodine deficiency was more pronounced in rural area (7.4%) than in urban area (3.6%).

In this study, urinary iodine status was also evaluated according to socio-economic characteristics and nutritional profile of recruited WRA. As reported in Table 4, no significant difference was observed for UIC and I/CR ratio according to the WRA age, their matrimonial status and educational level, as well as to the monthly household income (*p* > 0.05). Interestingly, a significant association was found between UIC and WRA nutritional status; obese WRA had the highest median value (83.7 μg/l), and UIC was higher in both thin and overweighed women than in normal weight women (*p* < 0.05). Moreover, results clearly showed that iodine deficiency is present in the 4 geographical origin with a median UIC ranging between 56.6 μg/l and 84.3 μg/l. Statistical analysis showed significant difference between iodine statutes, evaluated by UIC and I/CR, in the 4 regions (*p* < 0.05); lower UIC and I/CR medians being reported in zones 3 and 4 (Table 4).

**Table 4:** Urinary iodine concentration and iodine to creatinine ratio by nutritional and socio-economic characteristics of the studied population

	UIC	p value ^**a**^	I/Cr	p value ^**a**^
	Median [20th–80th]		Median [20th–80th]	
**Age**				
18–29	70.9 [37.5–118.6]	0.394	75.1 [43.5–141.6]	0.187
30–39	69.7 [35.8–122.2]		74.8 [43.5–153.1]	
40–49	74.5 [38.7–127.2]		80.8 [44.2–143.8]	
**Matrimonial status**				
Single	75.1 [38.3–116.4]	0.674	76.8 [46.1–140.0]	0.722
Married	70.6 [36.9–124.9]		76.6 [43.1–145.6]	
Divorced	73.7 [34.0–129.9]		71.9 [43.7–210.2]	
Widow	83.1 [46.6–105.6]		80.8 [52.7–164.9]	
**Educational level**				
Illiterate	69.7 [36.2–121.0]	0.081	74.8 [41.9–151.4]	0.629
Primary	74.6 [36.7–119.9]		79.4 [47.7–143.7]	
Secondary	67.3 [38.5–124.1]		76.0 [43.9–147.8]	
Superior	80.5 [39.6–132.6]		76.4 [49.7–136.8]	
**BMI (kg/m**^**2**^**)**				
Thinness	74.5 [44.0–110.9]	0.014	70.6 [45.6–136.8]	0.269
Normal weight	66.1 [34.2–118.4]		75.6 [44.1–143.9]	
Overweight	73.1 [36.7–121.9]		77.4 [43.5–149.9]	
Obese	83.7 [42.5–132.4]		79.4 [44.0–155.5]	
Monthly household income $ *				
⩽ 338	74.9 [39.1–129.7]	0.246	79.2 [47.1–150.2]	0.899
338–676	83.1 [41.8–132.1]		89.6 [46.9–175.8]	
677–1128	81.3 [40.7–115.2]		76.2 [49.5–170.9]	
⩾ 1129	61.1 [41.1–129.2]		84.4 [37.7–154.9]	
**Geographical origin**				
Zone 1	78.1 [39.5–134.3]	0.001	86.1 [48.4–164.7]	0.001
Zone 2	84.3 [47.4–134.1]		82.2 [51.2–158.9]	
Zone 3	56.6 [30.1–98.1]		65.4 [37.1–127.1]	
Zone 4	69.4 [39.9–131.8]		67.6 [41.3–138.1]	

^a^ *p*-value was calculated using the Kruskal Wallis test

* Calculated based on non-missing data (*n* = 1118)

**Fig. 2:**
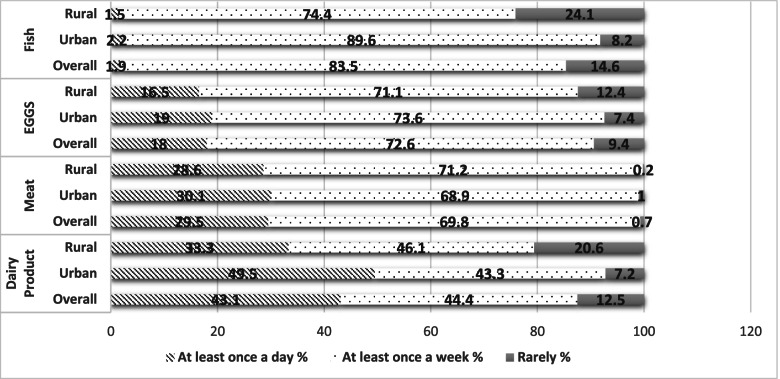
Percentages of consumption of iodine-rich food by the studied population overall and by rural and urban areas

In this study, the level of consumption of iodine-rich food, particularly fish, eggs, meat and dairy products, was also evaluated and results are reported in the Fig. 2. The most commonly consumed iodine-rich food was the group of dairy products that was reported to be consumed by 43.1% of WRA with a frequency of at least once a day. Meat and eggs were reported to be consumed daily by 29.5 and 18%, respectively, and weekly by 69.8 and 72.6%, respectively. Interestingly, 83.5% of WRA reported a weekly consumption of fish. Distribution of iodine-food consumption according to WRA living area showed a significant difference between rural and urban areas (*p* < 0.05). In fact, WRA from urban area consume more frequently fish and dairy products than WRA from rural areas. However, no significant difference was obtained for meat and eggs (*p* > 0.05), as they were practically consumed by WRA from both urban and rural areas.

## Discussion

During last decades, Morocco has adopted nutritional education and salt iodization to fight against iodine deficiency and to meet WHO target on increasing dietary iodine and thereby eliminating iodine deficiency. There’s currently an urgent need to assess the iodine status of Moroccan population to reinforce and/or to readjust the health nutrition program. To the best of our knowledge, the present study is the first one to be conducted on representative population from the whole country. This study was conducted as part of the national Stepwise 2017–2018 survey and was planned to provide the current iodine nutrition status in Moroccan WRA and to evaluate the prevalence of iodine deficiency in Morocco.

Iodine status is widely assessed by UIC determination that is highly useful in cross-sectional, epidemiological surveys in population samples of appropriate size. In this study, UIC was evaluated on spot urine samples, which have the obvious advantage to detect excess iodine intake with high sensitivity [9].

In the present study, the median UIC [20th–80th] was 71.3 μg/l [37.5–123.1] and 71% of recruited WRA have UIC less than 100 μg/l, indicating insufficient iodine status and confirming Morocco as a mild iodine deficiency country [9]. On the other hand, the prevalence of adequate iodine status (100–199 μg/l) was 22.1% whereas the prevalence of above adequate (299–200 μg/l) and excessive iodine status (≥300 μg/l) were 4.5% and 2,4%, respectively. This situation is of a particularly concern when comparing the median UIC with other neighbouring countries such as Algeria that has reported a median UIC of 253 μg/l in Tizi Ouzou and 256 μg/l in Algiers [35], and Spain that has reported a median UIC of 114 μg/l in a group of Spanish WRA [36]. Another study conducted on WRA from Sierra Leone has reported a median UIC of 203.3 μg/l [37].

A non-significant difference was observed for age, matrimonial status, educational level and monthly income categories (*p* > 0.05). However, a significant difference was found between the UIC values and WRA living areas: 63.40 μg/l [33.81–111.68] in rural area, versus 75.94 μg/l [41.16–129.97] in urban area (*p* < 0.05). This difference could be partly explained by the difference in eating habits, especially iodine-rich food consumption. In fact, the present study showed that women in rural area consume less frequently fish and dairy products, widely reported to be an important source of iodine. Difference between iodine status between rural and urban WRA is widely reported and documented and results are very controversial depending on the population and their eating habits and behaviours. In Iran, Machamba et al. have reported an iodine deficiency in women from rural areas and an excessive iodine in urban areas [38]. In contrast, the median UIC of Chinese rural residents was higher than that of urban residents [39].

In addition to the inadequate median iodine intake at the national level, the present study revealed large regional variation with evidence of high iodine deficiency in some areas. Iodine deficiency was more pronounced in the centre of the country, a non-coastal area characterized by a high altitude (Zone 3) and in the south part of the country regrouping costal, mountainous and desert regions (zone 4). This result is consistent with previous studies reporting that the prevalence of iodine deficiency increases with altitude and is more common among mountain dwellers [40, 41].

In Morocco, previous sporadic studies have evaluated iodine deficiency. In 1990s, the first regional iodine status monitoring revealed that 22% [95% CI: 20.0–24.1] of children (6–12 years) had a higher rate of goitre and reported a moderate deficiency with median iodine excretion of 86 μg/l, suggesting an overall low nutritional iodine status [42, 43]. An interesting study conducted by Aquaron et al. showed that in the mountain area of “Skoura-Toundoute”, the median UIC was very low, ranging from 18 to 24 μg/l, in both goitrous and non-goitrous patients [44]. Later, Zahidi et al. have reported an iodine deficiency in 94% of the population of Larache, a city in the Northern region of Morocco (63% moderate ID and 31% mild ID) [45]. Of particular interest, an UIC of 35 μg/l was reported in WRA from the Atlas Mountains of Southern Morocco [46]. It’s therefore clear that even the current situation is not satisfactory; it shows however a clear improvement of iodine status as compared to all previous reported data. This finding is corroborated with a recent data of the most recent national survey in Morocco on school-age children (6–12 years) that revealed adequate iodine status (mean UIC = 117.4 μg/l) and relative low iodine deficiency (UIC < 50 μg/l) that was reported in 21.6% [47]. These encouraging results are of a great purpose to strengthen much effort to fight against iodine deficiency in Morocco.

It’s largely accepted that iodine status is affected by the consumption of iodine-rich food. In the current study, results clearly showed that dairy products are the most commonly consumed iodine-rich food per day and per week in WRA. Scientific evidence clearly shows that dairy products are a good source of iodine and daily consumption of dairy products, particularly milk, is important for achieving adequate iodine status [48]. The iodine concentration in milk, and thus in all dairy products, is, however, dependent on the iodine status of the pastures. As a result, cows living in the inner land, including the center and mountainous areas, are more likely to produce iodine-deficient milk than cows living in coastal areas. Alternatively, to increase the productivity of iodine-enriched milk, careful consideration should be given to concentrating iodine in livestock feed. Forages, especially those near the sea, have a higher concentration of iodine, up to 0.2 mg/kg dry matter [49]. Interestingly, and for logistic consideration, fish consumption is more important in urban than rural areas.

This study is very informative and has much strength mainly the recruitment approach. In fact, this is the first study to be conducted on a nationally representative sample of Moroccan women of childbearing age. The study does, however, have some limitations: (1) as it is cross-sectional, it may not show the temporal relationship between the independent and dependent variables; (2) sources of dietary salt and household coverage of iodized salt were not assessed.

## Conclusion

Instead efforts made during last decades, Morocco is still a mildly iodine deficient country and iodine deficiency is yet a public health problem highlighting the necessity to implement effective national program in the different regions of Morocco, including efficient salt iodization, effective nutritional education and awareness to promote iodine-rich food consumption. Moreover, an effective monitoring program should be implemented to fellow iodine status and to prevent the population from developing iodine deficiency disorder.

## Data Availability

The data presented in this study are available on request from the corresponding author.

## Abbreviations

IDD: Iodine deficiency disorders
ID: Iodine deficiency
WRA: Women of reproductive age
IQR: Interquartile range
UIC: Urinary Iodine Concentration
UIE: Urinary iodine excretion
UI: Urinary iodine
I/CR: Iodine/Creatinine
WHO: World Health Organization
UNICEF: United Nations Children’s Fund
ICCIDD: International Council for Control of Iodine Deficiency Disorders
